# Spatiotemporal distribution and insecticide resistance status of *Aedes aegypti* in Ghana

**DOI:** 10.1186/s13071-022-05179-w

**Published:** 2022-02-19

**Authors:** Christopher M. Owusu-Asenso, Julius A. A. Mingle, David Weetman, Yaw A. Afrane

**Affiliations:** 1 Department of Medical Microbiology, University of Ghana Medical School, University of Ghana, Korle-Bu, Accra, Ghana; 2 Department of Vector Biology, Liverpool School of Tropical Medicine, Liverpool, UK

**Keywords:** *Aedes aegypti*, Insecticide resistance, Human blood index, Seasons, Ghana

## Abstract

**Background:**

Vector control is the main intervention used to control arboviral diseases transmitted by *Aedes* mosquitoes because there are no effective vaccines or treatments for most of them. Control of *Aedes* mosquitoes relies heavily on the use of insecticides, the effectiveness of which may be impacted by resistance. In addition, rational insecticide application requires detailed knowledge of vector distribution, dynamics, resting, and feeding behaviours, which are poorly understood for *Aedes* mosquitoes in Africa. This study investigated the spatiotemporal distribution and insecticide resistance status of *Aedes aegypti* across ecological extremes of Ghana.

**Methods:**

Immature mosquitoes were sampled from containers in and around human dwellings at seven study sites in urban, suburban, and rural areas of Ghana. Adult *Aedes* mosquitoes were sampled indoors and outdoors using Biogents BG-Sentinel 2 mosquito traps, human landing catches, and Prokopack aspiration. Distributions of immature and adult *Aedes* mosquitoes were determined indoors and outdoors during dry and rainy seasons at all sites. The phenotypic resistance status of *Aedes* mosquitoes to insecticides was determined using World Health Organization susceptibility bioassays. The host blood meal source was determined by polymerase chain reaction.

**Results:**

A total of 16,711 immature *Aedes* were sampled, with over 70% found in car tyres. Significantly more breeding containers had *Aedes* immatures during the rainy season (11,856; 70.95%) compared to the dry season (4855; 29.05%). A total of 1895 adult *Aedes* mosquitos were collected, including *Aedes aegypti* (97.8%), *Aedes africanus* (2.1%) and *Aedes* *luteocephalus* (0.1%). Indoor sampling of adult *Aedes* yielded a total of 381 (20.1%) and outdoor sampling a total of 1514 (79.9%) mosquitoes (*z* = − 5.427, *P* = 0.0000) over the entire sampling period. *Aedes aegypti* populations were resistant to dichlorodiphenyltrichloroethane at all study sites. Vectors showed suspected resistance to bendiocarb (96–97%), permethrin (90–96%) and deltamethrin (91–96%), and were susceptible to the organophosphate for all study sites. Blood meal analysis showed that the *Aedes* mosquitoes were mostly anthropophilic, with a human blood index of 0.9 (i.e. humans, 90%; human and dog, 5%; dog and cow, 5%).

**Conclusions:**

*Aedes* mosquitoes were found at high densities in all ecological zones of Ghana. Resistance of *Aedes* spp. to pyrethroids and carbamates may limit the efficacy of vector control programmes and thus requires careful monitoring.

**Graphical abstract:**

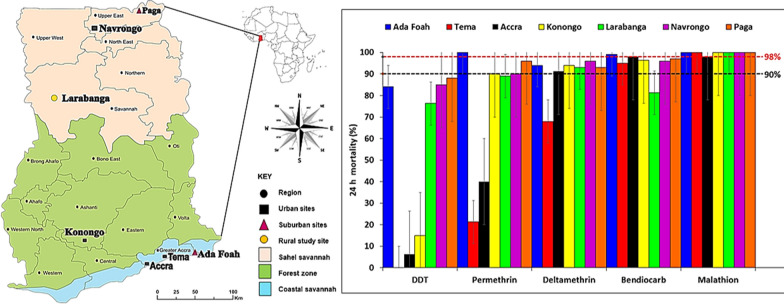

## Background

*Aedes aegypti* and *Aedes albopictus* are the most important vectors of several arboviruses, notably yellow fever, dengue, chikungunya, and Zika virus [1]. The importance of *Aedes* mosquitoes in sub-Saharan Africa has increased recently because of outbreaks of arboviral diseases in multiple countries there [2]. In West Africa, within the last 5 years, there have been outbreaks of dengue in Burkina Faso [3–5], Cote d’Ivoire [6, 7], and Senegal [8], yellow fever in Cote d’Ivoire [9–11] and Nigeria [12–16], and recent confirmed cases of dengue and outbreaks of yellow fever in Ghana [17–20]. Therefore, the risk of dengue, yellow fever and chikungunya outbreaks in Ghana appears to be high.

*Aedes aegypti* are highly anthropophilic and, throughout most of the world, typically endophilic [21]. Immature stages develop preferentially in artificial containers, usually in close proximity to humans [22–25]. In sub-Saharan Africa, two morphological subspecies (ecotypes) have been acknowledged: domestic *Aedes aegypti aegypti* and sylvan *Aedes aegypti formosus*. The presence/absence of white abdominal scale patterns [26] is used to differentiate the ecotypes, but at present, clear genetic boundaries appear to be absent, probably as a result of recent and historical gene flow [27, 28]. *Aedes aegypti formosus* more frequently breed away from domestic environments, and feed readily on animals (zoophagy), so are less likely to be a threat to humans in the urban environments where *Aedes aegypti aegypti* populations thrive [29]. However, urbanisation of the sylvatic environment could lead to contact between *Ae. aegypti formosus* and humans, and this typically sylvatic species might adapt to new urban environments and hosts; it is also probable that introgression through hybridization of urban and sylvatic subspecies of *Ae. aegypti* may lead to variation, potentially increasing the role of *Ae. aegypti formosus* as vectors [28, 30]. The consistency of bionomic traits across ecozones remains poorly investigated. However, measures of abundance and distribution of *Aedes* would give more reliable insights for both risk and mitigation strategies for infestations [31]. Several species of *Aedes*, including *Aedes africanus*, readily feed on animals (both domestic and wild), as well as humans, hence their potential importance as bridge vectors and for zoonotic transmission [32]. Identification of the source of vector blood meals is critical to understanding the degree of human–vector interaction (i.e. anthropophily) [33, 34], which is a crucial parameter in the estimation of the capacity of a vector to transmit a disease [35].

Seasonal variations in population density are expected for *Aedes*, with lower abundances in dry seasons, rising with increasing temperatures, and potentially greater breeding site availability in the rainy season [36–38]. However, human activities involving water storage and the disposal of potential water-holding containers greatly influence the breeding of *Aedes* in individual households and may lead to the provision of breeding sites year-round [39]. Key factors that may influence *Aedes* productivity in different container types include the frequency of water replenishment, the availability of food for the larvae, the degree of sunlight exposure [40], and container coverings [41]. The adaptation of these vectors to urban domestic habitats has led to their exploitation of a range of artificial containers and their capacity to exploit potential breeding water situated indoors or outdoors [42, 43].

Currently, and despite frequent concerns regarding the efficacy of the methods used for their deployment [44], insecticidal interventions are the main tool used to control *Aedes*-borne arboviral infections, since vaccines for these are either unavailable, ineffective, or in limited supply [45–47]. To ensure that efficacy is maximised, correct insecticide choice is crucial, and requires surveillance to determine the susceptibility of target populations, alongside locating the adults and immatures that are to be targeted [48]. Sustained effectiveness must also be considered: geographical variation in susceptibility may rapidly lead to the spread of insecticide resistance and require revision regarding the most suitable insecticide for use.

Another important parameter when considering how to target vector control, especially for insecticidal spray deployment, is whether mosquitoes tend to rest indoors (endophily) or outdoors (exophily) after blood-feeding [49]. Insecticide-based intervention directed at the adult resting population is a relevant approach for *Aedes* control and disease prevention. Targeted indoor residual spraying on *Aedes* resting locations can provide a significant protective effect against arboviral transmission, and this method also has the potential to control pyrethroid-resistant *Aedes* mosquitoes, as other classes of insecticides (non-pyrethroids) are available for residual application [50].

This study aimed to characterize the breeding habitats, seasonal abundance, and resting behaviour of *Ae. aegypti*, and their insecticide susceptibility, in rural, suburban, and urban sites in different ecological zones of Ghana. In addition to identifying targets and options for control, the results will also aid the development of a surveillance system for *Aedes* as vectors of arboviruses for the planning of disease control in Ghana [51, 52].

## Methods

### Study sites

**Fig. 1:**
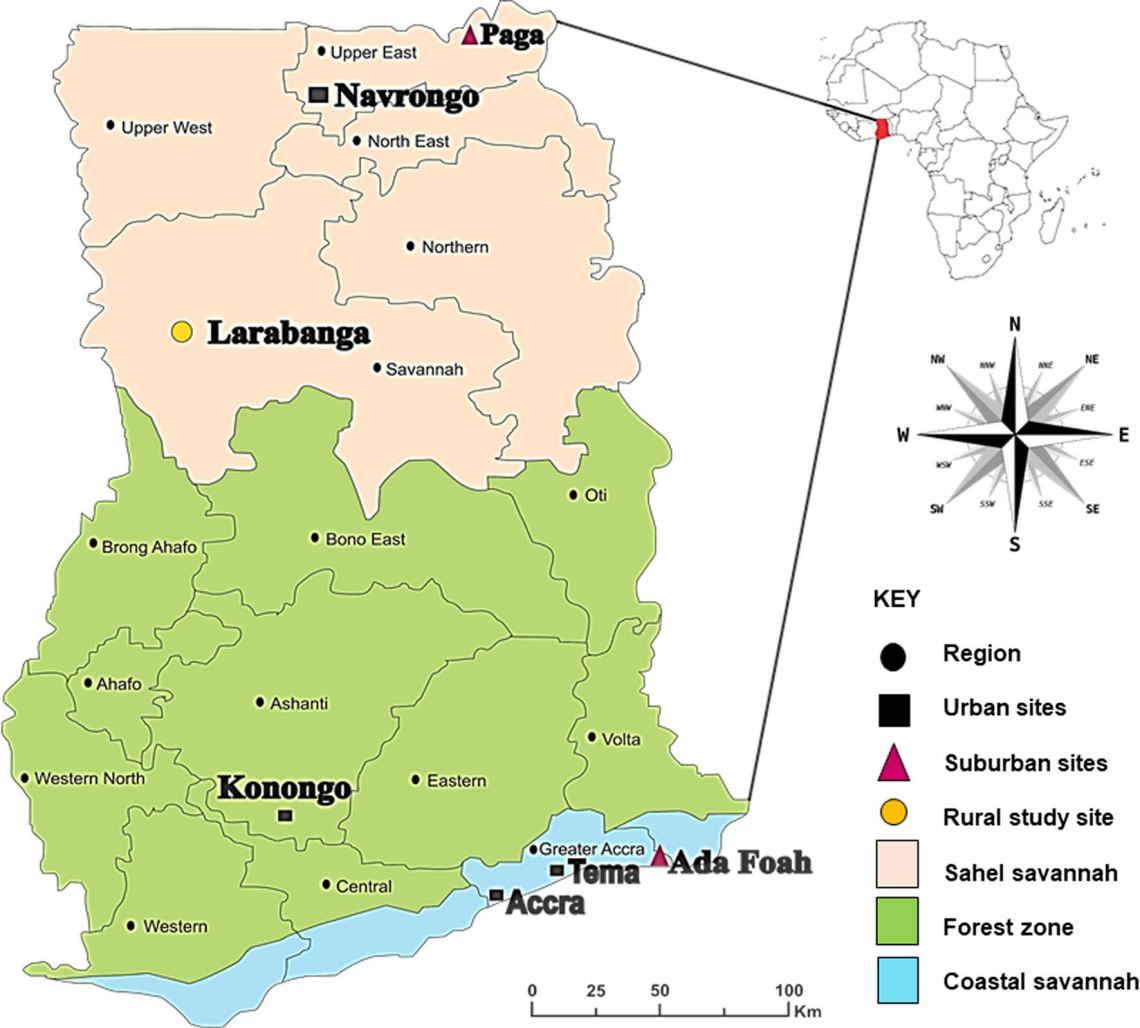
Map of Ghana showing the study sites

This study was carried out in seven sites comprising rural, suburban, and urban locations within the three major ecological zones of Ghana, i.e. coastal savannah, forest, and Sahel savannah, across wet and dry seasons, between May 2017 and May 2018. The selection of the study sites was based on both ecological zone and population (urban, suburban, and rural), since variations in species, abundance, and susceptibility status of *Aedes* mosquitoes could be influenced by these parameters. Residents in the rural study sites store water for domestic use in diverse containers because the water supply system is unreliable. Water supply in the rural study sites is mainly from harvested rainwater, wells, and boreholes, and these also supplement the irregular piped water supply system characteristic of our urban and suburban study sites. Furthermore, it is known that *Ae. aegypti* is adapted to urban settings while *Ae*. *albopictus* and other zoonotic species are found in the sylvatic zone. One of the sites has a port where car tyres are imported, thus may be a channel for the importation of species of *Aedes* from the Americas previously unknown in Ghana. The sample sites are shown in Fig. 1.

In the coastal savannah zone of southern Ghana the study sites were as follows: Ada Foah (suburban site; 5°47′N, 0°38′E), a tourist town; Accra (urban site; 5°33′0″N, 0°12′0″W), the capital of Ghana and its most populous city; and Tema (urban site; 5°40′0″N, 0°0′0″E), a port city where the importation of tyres from Asia and the Americas might facilitate the invasion of *Aedes* genotypes or species previously unknown in Ghana. The coastal savannah has a tropical savannah climate, with an annual mean temperature of 26.5 °C and average annual precipitation of 787 mm (Table 1).

**Table 1:** Study site locations

Population	Major ecological zones of Ghana		
	Costal savannah	Forest zone	Sahel savannah
Urban	Accra (5°33′0″N, 0°12′0″W)	Konongo (06°37′00″N, 01°13′00″W)	Navrongo (10°53′5″N, 01°05′25″W)
	Tema (5°40′0″N, 0°0′0″E)		
Suburban	Ada Foah (5°47'N, 0°38'E)		Paga (10°59′32″N, 01°06′48″W)
Rural			Larabanga (9°5′0″N, 1°49′0″W)

The urban site within the forest zone was Konongo (06°37′00″N, 001°13′00″ W), a town located in Asante-Akim central district in the middle of Ghana. In the forest zone, there is a high possibility that sylvan *Aedes* mosquitoes, which can serve as bridge vectors, might be present. The forest zone has a tropical rainforest climate, with an annual average temperature of 26.4 °C and annual average precipitation of 1399.5 mm (Table 1).

The sites in the Sahel savannah ecological zone were Larabanga, Navrongo, and Paga. Larabanga (rural site; 9°5′0″N, 1°49′0″W) is a village situated close to Mole national park, which harbours monkeys that could serve as reservoirs for arboviruses, and has experienced yellow fever outbreaks [53]. Navrongo (urban site; 10°53′5″N, 01°05′25″W) is a town close to the border between Ghana and Burkina Faso; an outbreak of dengue fever was relatively recently reported in Burkina Faso, in the period between 2016 and 2019 [54]. The last site was Paga (suburban site; 10°59′32″N, 01°06′48″W), a small town located on the border of Burkina Faso and 166 km south of Ouagadougou, where an outbreak of dengue fever was recently reported [54] (Table 1).

In both the coastal savannah and forest area there is generally a bimodal pattern of rainfall, with the long rainy season from April to June, and a short rainy season from October through November. Rainfall in the Sahel savannah is unimodal, with the rainy season between May and November and the dry season from December to April. Sampling was done during the rainy season from April through June 2017 in the coastal savannah and forest zone, from May through June 2018 in the Sahel savannah, during the dry season from January through March 2018 in the coastal savannah and forested savannah, and from December 2017 through January 2018 in the Sahel savannah.

### Distribution of immature *Aedes* mosquitoes

Exhaustive entomological surveys were carried out at each of the study sites. Water-holding containers in and around human dwellings were inspected for immature *Aedes* in the dry and rainy seasons, and those positive for *Aedes* immatures recorded. Larval habitats were sampled once per season. Every possible *Aedes* breeding container was inspected for the presence of *Aedes* immatures at each site. Because it was difficult to sample most of the containers in which the *Aedes* bred by dipping, all *Aedes* immatures encountered at each breeding habitat were collected to determine the density of vectors. All pupae and larvae (first to fourth instars) from positive containers (air conditioner saucers, car tyres, drums, tanks, buckets, and discarded containers) were collected using pipettes and ladles [39, 55], counted, and recorded on field data forms. Water from large containers was first sieved and larval samples placed in a white plastic tray with some of the water in which they were pipetted. Mosquito samples were placed immediately in labelled specimen bowls filled with water from the container from which they had been collected, and transported to the insectary. Immature mosquitoes were reared in the insectary in large white plastic trays at an average temperature of 28.15 ± 1.8 °C (± SD) and relative humidity of 80.9 ± 6.3%, and larvae were fed on TetraMin Baby fish food (Tetra Werke, Melle, Germany).

Adult female *Aedes* mosquitoes that emerged from the collected larvae were used for the World Health Organization (WHO) susceptibility bioassays [56] and later identified morphologically using standard taxonomic keys [57]. Coordinates of all collection points were recorded using a GPSMAP 60CSx Geographical Positioning System (GPS) instrument (Garmin International, Olathe, KS).

### Characterization of *Aedes* breeding habitats and relative abundance

**Fig. 2:**
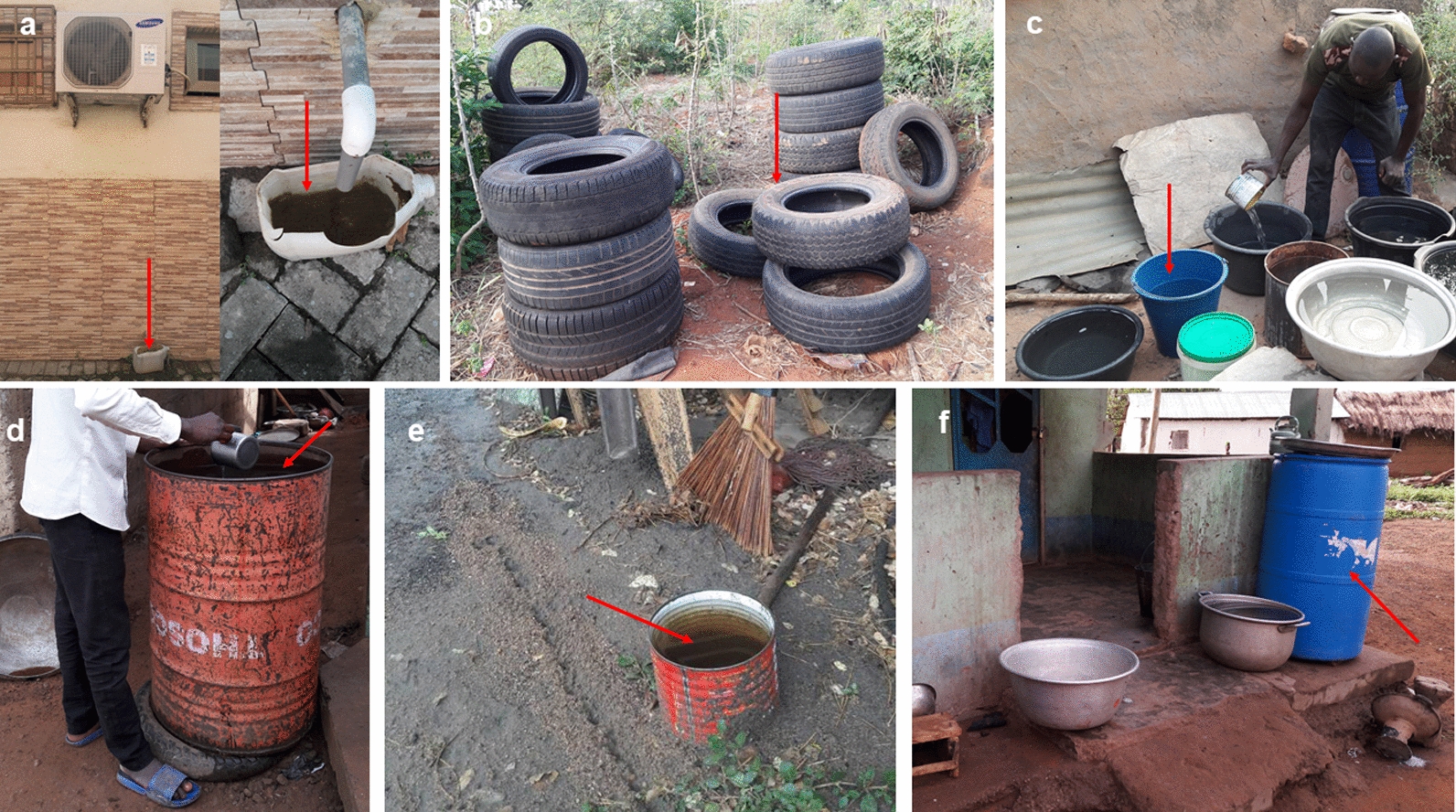
Habitat types encountered during the larval survey and sampling: **a** air conditioner saucer, **b** car tyres, **c** buckets, **d** tank, **e** discarded container, and **f** drum

For each entomological survey, the habitat type, its household location (indoors or outdoors), and its physical characteristics were recorded. Six container types were classified based on their use and material: car tyres, air conditioner saucers, discarded containers, drums, tanks, and buckets. Air conditioner saucers are small (1–2 L) plastic containers positioned below the outlet of air conditioners to collect water. Discarded containers were defined as 50- to 100-L-capacity containers, which included broken jars, bottles, small plastic food containers, tins, plates, cans, cooking pots, and broken pots made of plastic or metal. Drums were defined as 100- to 500-L-capacity plastic water storage containers. Tanks were defined as 100- to 500-L-capacity water storage containers made of metal or concrete. Buckets comprised 10- to 25-L water storage containers made of metal or plastic. It is notable that pipeborne water was absent in Larabanga and Paga, with the consequence that households tend to have long-term water storage in tanks, drums, buckets, and pots, especially during the dry season, which may serve as potential *Aedes* larval breeding habitats (Fig. 2).

### Distribution of adult *Aedes* mosquitoes

The spatial distribution of adult *Aedes* mosquitoes was determined by sampling inside households and outdoors. In the Sahel savannah zone, *Aedes* mosquitoes were sampled indoors and outdoors using three methods that employed one of the following: BG-Sentinel 2 traps (BG traps; Biogents, Regensburg, Germany); human landing catches (HLC); or Prokopack Aspirators (PPK) (Hock, Gainesville, FL) [58]. The relative trapping efficiency of the three sampling methods, i.e. using BG traps, HLC, or PPK, was compared for the Sahel savannah zone to determine the most efficient vector sampling tool for future surveillance. Due to logistical challenges, in the coastal and forest ecological zones, only two of the methods were employed, the one which used BG traps and the one which used HLC.

The GPS coordinates of all collection points were recorded. Two cross-sectional surveys were undertaken, one in the dry season (December 2017–March 2018) and one in the rainy season (April–June 2017 and May–June 2018).

### Adult mosquito collection using BG traps, HLC and PPK

BG traps were set both indoors [living room(s) and bedrooms] and outdoors (open but secure verandas, granaries, or under a shed/tree where people sit to chat, about 5 m from the house) from 5:00 to 8:00 a.m. and 3:00–7:00 p.m. The BG traps were baited with CO_2_ which was produced by either BG-Lures and/or from a mixture of 17.5 g yeast [Angel Yeast (Egypt)] and 250 g sugar in 1 L of water [59]. The mosquito collection net of the BG trap was changed at 1-h intervals. Mosquitoes trapped within the collection net were placed in a cooler box containing ice and then transported to the insectary.

The HLC method was also used to sample host-seeking adult *Aedes* mosquitoes. On each day, two trained volunteers were positioned indoors and two were positioned outdoors to catch *Aedes* mosquitoes. The collected *Aedes* were placed in labelled paper cups, which were placed in cool boxes with ice packs and transported to the insectary for identification and further processing.

Prokopack aspiration was employed at the three sites in the Sahel savannah area: Larabanga, Navrongo, and Paga. Sixteen houses were randomly selected for *Aedes* collection per site. *Aedes* mosquitoes were sampled indoors and outdoors. *Aedes* caught within the Prokopack plastic collection cups were labelled and placed in a cooler box containing an ice pack and transported to the insectary for identification. The heights at which the mosquitoes were caught by PPK while resting were recorded using a tape measure, to determine whether there was heterogeneity in resting height among sites.

Sixteen houses were randomly selected for each sampling method at each site. Sampling using each type of sampling tool was done on 4 different days during each season. On each of the sampling days, the houses used for sampling had not previously been used for this purpose. Mosquito sample collection using PPK and HLC was done hourly from 5:00 to 8:00 a.m. and then from 3:00 to 7:00 p.m.

Meetings were held at each study site with chiefs and residents to introduce the research study to the public. All participants in this study were adults (> 18 years old). Written and signed consent was obtained from all of the adults who volunteered to participate in HLC before they were trained and the mosquito sampling began. A copy of the signed consent form was given to each of the HLC volunteers and another copy kept in a locked cabinet with restricted access in the offices of the Department of Medical Microbiology, University of Ghana Medical School. Verbal and written consent was obtained from household heads to sample mosquitoes in their houses and compounds. All volunteers were remunerated at the end of the study.

### Insecticide susceptibility tests

*Aedes* larvae were collected from natural breeding sites or from oviposition traps that were set in each site. Oviposition traps were made from used car tyres that had been cut into three parts which could hold about 3 L of water each. Oviposition traps were set for the collection of *Aedes* immatures during time points when it was difficult to obtain sufficient numbers for the WHO susceptibility bioassays. Collected larvae were brought to the insectary at the Department of Medical Microbiology, University of Ghana, and were raised to adults under standard conditions (25 ± 2 °C, 80% ± 4% relative humidity, 12-h:12-h light/dark cycle). Batches of 20–25 non-blood-fed 3- to 5-day-old females were used for the susceptibility bioassays. Four replicates and two controls were used for each insecticide tested using the standard WHO susceptibility bioassay procedure [56].

 The WHO test papers were impregnated with a pyrethroid (0.05% deltamethrin or 0.75% permethrin), an organochloride [4% dichlorodiphenyltrichloroethane (DDT)], an organophosphate (5% malathion), or a carbamate (0.1% bendiocarb) insecticide. These pre-impregnated papers are supplied with diagnostic concentrations based on those required for *Anopheles*. As the concentrations of permethrin and malathion of the test papers are three times and approximately six times, respectively, the diagnostic concentrations required for *Aedes* mosquitoes, this was a limitation of the study. However, these test papers are far more commonly used for the assessment of the susceptibility of *Aedes* to these insecticides than those custom produced at the recommended concentrations [60]. The knockdown time was reported every 10 min during the 60-min exposure period. Mortality was recorded after the 24-h recovery period. ‘Resistant’ mosquitoes were defined as mosquitoes that survived for 24 h after the end of the bioassay, and ‘susceptible’ mosquitoes as those that were knocked down or died during the 60-min exposure time, or that died within the 24-h recovery period.

### Data analysis

Descriptive analysis was performed to compare larval and adult abundance between different populations (urban, suburban and rural), indoors and outdoors, and seasons.

The abundances of *Aedes* larvae and adults were compared among the seasons, indoor and outdoor study sites (ecozones), and sampling methods (adults). For all sites, a Kruskal–Wallis test was used to compare the abundance of adult mosquitoes for HLC and BG traps, and a Wilcoxon rank-sum test was used to test for associations between continuous and categorical variables. Nested generalized linear mixed models with sites nested within ecological zones were used to model the effect of ecozone, season, population (urban, suburban and rural), and sampling methods on larval and adult abundance. A regression analysis was done to test trap efficacy. Probability values less than 0.05 were interpreted as statistically significant.

Human blood index was calculated as the proportion of positive human blood specimens per total number of specimens tested.

Insecticide susceptibility was classified using the following WHO criteria [56]: 98–100% mortality, the test population is considered susceptible; 90–97% mortality, possible resistance of the test population (which requires confirmation); below 90% mortality, the test population is considered resistant. Knockdown and mortality rates were compared between sites using Chi-square. Statistical analysis was performed using Stata 16 (StataCorp, College Station, TX).

## Results

### Larval breeding habitats and their productivity

A total of 81 positive breeding habitats were identified during the study period across the seven sites (only positive breeding habitats were recorded). Generalized linear model analysis revealed a significant interaction effect between ecozone and population on abundance. Compared to the other sites, the chance of sampling *Aedes* larvae was higher in the forest zone [unadjusted (unadj.) *B* = − 204.12 (− 306.01 to − 102.24), *P* = 0.000]. The abundance of *Aedes* larvae was higher in suburban areas [unadj. *B* = − 138.01 (− 224.77 to − 51.26), *P* = 0.002] than in urban areas (Table 2).

**Table 2:** Factors associated with the productivity of larval habitats and larval abundance

Characteristics	Category	Unadjusted *B* (CI)	*P-*value	Adjusted* B *(CI)	*P*-value
Season	Dry	1		1	
	Rainy	80.51 (− 2.26 to 163.27)	0.057	65.22 (− 12.02 to 142.46)	0.098
Ecozone	Coastal savannah	1		1	
	Forest	− 204.12 (− 306.01 to − 102.24)	0.000	− 184.81 (− 358.62 to − 10.99)	0.037
	Sahel savannah	− 72.9569 (− 157.57 to 11.65)	0.091	− 61.23 (− 158.68 to 36.21)	0.218
Indoors/outdoors	Indoors	1		1	
	Outdoors	61.94 (− 155.38 to 279.25)	0.576	30.59 (− .194.75 to 255.92)	0.790
Population	Urban	1		1	
	Suburban	8.62 (− 96.67 to 113.90)	0.873	13.89 (− 88.91 to 116.71)	0.791
	Rural	− 138.01 (− 224.77 to − 51.26)	0.002	− 9.43 (− 156.49 to 137.63)	0.900

*CI* Confidence interval

There were significantly more positive habitats during the rainy season than during the dry season (*n* = 50 vs *n* = 31; *df* = 5, χ^2^ = 19.44, *P* = 0.001; Table 3). Within the seven sites sampled, 78 (96.3%) of the larval breeding habitats were located outdoors and three (3.7%) indoors (all in Larabanga) (Table 3), with larval abundances of 16,426 (98.3%) and 285 (1.7%), respectively (*n* = 78 vs *n* = 3; *z* = − 0.138, *P* = 0.8903).

**Table 3:** Seasonal distribution of positive breeding habitats by location and season

Container type	Season		Location		Total (%)
	Dry (%)	Rainy (%)	Indoors (%)	Outdoors (%)	
Tyre	26 (44.07)	33 (55.93)	0	59	59 (100.00)
Container	0	15 (100.00)	0	15	15 (100.00)
Bucket	2 (100.00)	0	2	0	2 (100.00)
Tank	0	1 (100.00)	0	1	1 (100.00)
Drum	0	1(100.00)	1	0	1 (100.00)
Air conditioner saucer	3 (100.00)	0	0	3	3 (100.00)
Total	31 (38.27)	50 (61.73)	3 (3.7)	78 (96.3)	81 (100.00)

A total of 16,711 *Aedes* immatures were collected over the entire sampling period, of which 12,348 (73.9%) were from car tyres, 3138 (18.8%) from discarded containers, 730 (4.4%) from air conditioner saucers, 230 (1.4%) from buckets, 210 (1.3%) from tanks, and 55 (0.3%) from drums (χ^2^ = 1.020, *df* = 5, *P* = 0.96; Table 4). For all the different sites, car tyres had the highest proportion, 71.3% (8453), of immatures during the rainy season. The same observation was made during the dry season, with the highest abundance of *Aedes* immatures, 3895 (80.2%) (χ^2^ = 2.106, *df* = 2, *P* = 0.3490; Table 4), in car tyres.

**Table 4:** Productivity profile (number of *Aedes* immatures) of container type per site and season

Container type	Season	Ada Foah	Tema	Accra	Konongo	Larabanga	Navrongo	Paga
Car tyres	Dry	695	2260	535	0	405	0	0
	Rainy	1066	2078	487	558	505	1680	2079
Discarded containers	Dry	0	0	0	0	0	0	0
	Rainy	50	0	1918	540	540	90	0
Air conditioner saucer	Dry	0	0	730	0	0	0	0
	Rainy	0	0	0	0	0	0	0
Bucket	Dry	0	0	0	0	230	0	0
	Rainy	0	0	0	0	0	0	0
Tank	Dry	0	0	0	0	0	0	0
	Rainy	0	0	0	0	210	0	0
Drum	Dry	0	0	0	0	0	0	0
	Rainy	0	0	0	0	55	0	0
Seasonal totals	Dry	695	2260	1265	0	635	0	0
	Rainy	1116	2078	2405	1098	1310	1770	2079
Total		1811	4338	3670	1098	1945	1770	2079

Regarding the different ecological zones, significantly more *Aedes* immatures were collected from the coastal savannah (9819; 58.8%), followed by the Sahel savannah (5794; 34.7%), and then the forest zone (1098; 6.6%) (χ^2^ = 16.071, *df* = 2, *P* = 0.0003). A higher proportion of immature *Aedes* was collected in urban areas, with an abundance of 10,876 (65.1%) (Tema, 4338; Accra, 3670; Navrongo, 1770; Konongo, 1098), followed by the suburban areas, with a total of 3890 (23.3%) (Paga, 2079; Ada Foah, 1811), and then rural areas, with a total abundance of 1945 (11.6%) (Larabanga, 1945) (χ^2^ = 10.040, *df* = 2, *P* = 0.0066). We found more *Aedes* immatures outdoors (16,426; 98.3%) than indoors (285; 1.7%) (*z* = − 0.138, *P* = 0.8903).

### Seasonal distribution of adult *Aedes* mosquitoes

A total of 1895 adult *Aedes* mosquitoes were collected from the study sites. Generalized linear model analysis revealed a significant interaction effect between outdoor collection, ecozone and population (urban, suburban, and rural) on abundance. The chance of sampling adult *Aedes* mosquitoes increased outdoors [adj. *B* = 1.49 (1.0271–1.9602), *P* = 0.000]. There was a significant difference between adult *Aedes* mosquito abundance in suburban sites [adj. *B* = − 1.49 (− 2.0433 to − 0.9320), *P* = 0.000] and urban sites (Table 5). Adult *Aedes* were more abundant during the rainy season (1257; 66.3%) compared to the dry season (638; 33.7%) (*z* = − 1.433, *P* = 0.1519). Across the different ecological zones, the abundance of *Aedes* was high in the coastal savannah (955; 50.4%) [Accra (urban), 718; Tema (urban), 161; Ada Foah (suburban), 76], followed by the Sahel savannah 837 (44.2%) [Navrongo (urban), 577; Paga (suburban), 173; Larabanga (rural), 87], and the forest zone (103; 5.4%) [Konongo (urban), 103] ($\chi$^2^ = 0.359, *df* = 2, *P* = 0.835). The urban sites had the highest abundances of *Aedes* mosquitoes (1559, 82.3%) (Accra, 718; Tema, 161; Konongo, 103; Navrongo, 577) followed by the suburban sites (249; 13.1%) (Ada Foah, 76; Paga, 173), and then the rural site (87; 4.6%) (Larabanga, 87) ($\chi$^2^ = 20.147, *df* = 2, *P* = 0.0001).

**Table 5:** Factors associated with adult *Aedes* mosquito abundance

Characteristics	Category	Unadjusted *B* (CI)	*P-*value	Adjusted *B* (CI)	*P*-value
Season	Dry	1		1	
	Rainy	0.34 (− 0.1487 to 0.8221)	0.174	0.49 (− 0.0109 to 0.9931)	0.055
Ecozone	Coastal savannah	1		1	
	Forest	− 0.44 (− 1.3873 to 0.5069)	0.362	− 1.16 (− 2.1213 to − 0.2186)	0.016
	Sahel savannah	− .16 (− 0.6561 to 0.3312)	0.519	0.19 (− 0.3609 to 0.746917)	0.495
Indoors/outdoors	Indoors	1		1	
	Outdoors	1.45 (0.9840–1.9242)	0.000	1.49 (1.0271–1.9602)	0.000
Population	Urban	1		1	
	Suburban	− 1.25 (− 1.8001 to − 0.7042)	0.000	− 1.49 (− 2.0433 to − 0.9320)	0.000
	Rural	− 1.49 (− 2.1720 to − 0.8260)	0.000	− 1.844 (− 2.5952 to − 1.0935)	0.000

At the different sites during the dry season, the highest abundance of *Aedes* mosquitoes was found in Accra (178; 27.9%) (HLC, 163; BG trap, 15), followed by Navrongo (173; (27.1%) (HLC, 157; BG trap, 16), Tema (108; 16.9%) (HLC, 102; BG trap, 6), Konongo (103; 16.1%) (HLC, 88; BG trap, 15), Ada (60; 9.4%) (HLC, 50; BG trap, 10), Larabanga (15; 2.4%) (HLC, 0; BG trap, 15), and then Paga (1; 0.2%) (HLC, 1; BG trap, 0) ($\chi$^2^ = 20.500, *df* = 6, *P* = 0.0023).

During the rainy season, the highest abundance of *Aedes* mosquitoes was found in Accra (540; 43.0%) (HLC, 499; BG trap, 41), followed by Navrongo (404; 32.1%) (HLC, 354; BG trap, 50), Paga (172; 13.7%) (HLC, 168; BG trap, 4), Larabanga (72; 5.7%) (HLC, 54; BG trap, 18), Tema (53; 4.2%) (HLC, 31; BG trap, 22), Ada Foah (16; 1.3%) (HLC, 0; BG trap, 16), and then Konongo (0, 0%) (HLC, 0; BG trap, 0), ($\chi$^2^ = 132.896, *df* = 6, *P* = 0.0001).

### Indoor and outdoor abundance of adult *Aedes* populations

**Fig. 3:**
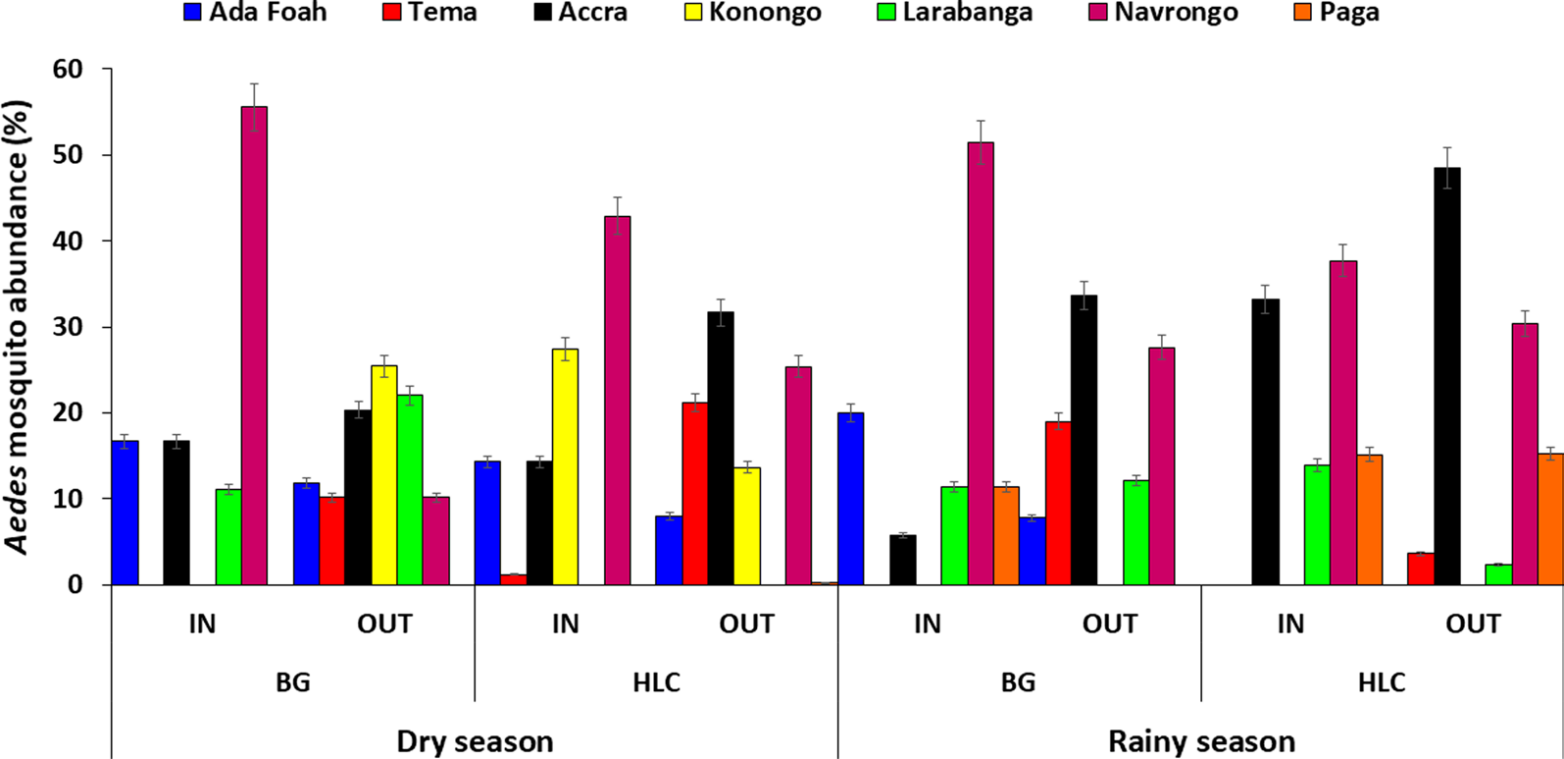
Seasonal distribution of adult *Aedes* mosquitoes captured indoors (*IN*) and outdoors (*OUT*). *BG* BG-Sentinel 2 trap,* HLC* human landing catches

Overall mosquito abundance was highest outdoors as compared to indoors over the entire sampling period. Indoor sampling yielded a total of 381 (20.1%) and outdoor sampling a total of 1514 (79.9%) adult *Aedes* over the entire sampling period (*z* = − 5.427, *P* = 0.0000). During the rainy season, a higher proportion of *Aedes* mosquitoes was captured outdoors (77.8%; 978) than indoors (22.2%; 279) (*z* = − 2.989, *P* = 0.0028). Similarly, a greater number of *Aedes* mosquitoes were captured outdoors (536; 84%) than indoors (102; 16%) during the dry season (*z* = − 5.021, *P* = 0.0000; Fig. 3).

### Comparison of trap efficiency for *Aedes* mosquito sampling

A total of 1140 *Aedes* mosquitoes were collected by HLC, BG traps, and PPK in Larabanga, Navrongo, and Paga during the experiment. Overall, the abundance of adult *Aedes* mosquitoes was 2.4 times higher for HLC (734) compared to PPK (303), and 7.1 times higher for HLC compared to BG traps (103). There was a significant difference between the abundance of *Aedes* mosquitoes for HLC and BG traps [*P* = 0.000, 95% confidence interval (CI) = − 2.180248 to − 0.7245137], but no significant difference between HLC and PPK (*P* = 0.350, 95% CI = − 0.4820909 to 1.359284; Table 6).

**Table 6:** Comparison of sampling methods for the collection of mosquitoes in the Sahel ecological zone

Study sites	Dry season						Rainy season					
	BG trap (%)		HLC (%)		PPK (%)		BG trap (%)		HLC (%)		PPK (%)	
	In.	Out.	In.	Out.	In.	Out.	In.	Out.	In.	Out.	In.	Out.
Larabanga	2 (13.3)	13 (86.7)	0	0	1 (4.4)	22 (95.6)	4 (22.2)	14 (77.8)	34 (63)	20 (37)	40 (32)	86 (68)
Navrongo	10 (62.5)	6 (37.5)	36 (22.9)	121 (77.1)	0	22 (100)	18 (36)	32 (64)	92 (26)	262 (74)	0	8 (100)
Paga	0	0	0	1 (100)	0	0	4 (100)	0	37 (22)	131 (78)	87 (70)	37 (30)
Total	12 (5.1)	19 (8.1)	36 (15.4)	122 (52.1)	1 (0.4)	44 (18.8)	26 (2.8)	46 (5.1)	163 (18)	413 (45.6)	127 (14)	131 (14.5)
Total per trap	31 (2.7)		158 (13.9)		45 (3.9)		72 (6.3)		576 (50.5)		258 (22.6)	

*BG trap* BG-Sentinel 2 trap,* HLC* human landing catch,* PPK* Prokopack Aspirator, *In*. indoors,* Out*. outdoors

Generalized linear model analysis revealed a significant interaction effect between outdoor collection, study site and sampling method on abundance. More adult *Aedes* mosquitoes were collected outdoors [adj. *B* = 0.87 (0.22, 1.52), *P* = 0.009]. Adult *Aedes* mosquitoes were more abundant in Navrongo [adj. *B* = 0.83 (0.07, 1.58), *P* = 0.032], and BG traps were the least efficient means of collecting *Aedes* mosquitoes [adj. *B* = − 1.39 (− 2.14, − 0.64), *P* < 0.001; Table 7].

**Table 7:** Factors associated with the capture efficiency of HLC, BG traps, and PPK

Characteristics	Category	Unadjusted *B* (CI)	*P-*value	Adjusted *B* (CI)	*P*-value
Season	Dry	1		1	
	Rainy	0.31 (− 0.45, 1.08)	0.425	0.09 (− 0.71, 0.89)	0.821
Indoors/outdoors	Indoors	1		1	
	Outdoors	0.90 (0.24, 1.56)	0.007	0. 87 (0.22, 1.52)	0.009
Site	Larabanga	1		1	
	Navrongo	0.98 (0.22, 1.74)	0.011	0.83 (0.07, 1.58)	0.032
	Paga	0.65 (− 0.22, 1.52)	0.142	0.34 (− 0.54, 1.23)	0.446
Trap	HLC	1		1	
	BG trap	− 1.45 (− 2.18, − 0.72)	0.0001	− 1.39 (− 2.14, − 0.64)	0.000
	PPK	0.44 (− 0.48, 1.36)	0.350	0.42 (− 0.50, 1.34)	0.373

For abbreviations, see Tables 2 and 6

### Resting height of *Aedes* mosquitoes

The maximum height at which *Aedes* mosquitoes were caught resting was 5 m and the lowest 1 m. The mean preferred resting height of the caught *Aedes* mosquitoes ranged from 1.8 to 2.0 m indoors and 1.3–2.8 m outdoors ($\chi$ ^2^ = 1.408, *df* = 2, *P* = 0.4945). No mosquito was caught resting indoors in Navrongo (Table 8).

**Table 8:** Resting heights of *Aedes* per study site

Study site	Total number of mosquitoes (%)	Total number of mosquitoes indoors	Total number of mosquitoes outdoors	Highest height of houses (m)	Average mosquito resting height (indoors) (m)	Average mosquito resting height (outdoors) (m)
Paga	124 (48.1)	87	37	4	1.8	1.3
Navrongo	8 (3.1)	0	8	5	–	2.8
Larabanga	126 (48.8)	40	86	5	2.0	2.4

### *Aedes* species composition at the study sites

Morphological identification of all collected adult *Aedes* showed that *Aedes aegypti* (1854; 97.8%) was the most abundant species present at all sites followed by *Aedes africanus* (40; 2.1%) and *Aedes luteocephalus* (1; 0.01%) (Table 9). All 11,506 *Aedes* mosquitoes that emerged from the larvae collected from the sites and reared in the insectary were identified morphologically as *Aedes aegypti* (Table 9).

**Table 9:** Number of *Aedes* mosquitoes per study site identified morphologically to species level

Study site	Adults				Larvae^a^
	Total per site (%)	*Aedes aegypti* (%)	*Aedes africanus* (%)	*Aedes luteocephalus* (%)	*Aedes aegypti* (%)
Ada Foah	76 (100.0)	64 (84.2)	12 (15.8)	0 (0.0)	981 (100.0)
Tema	161 (100.0)	161 (100.0)	0 (0.0)	0 (0.0)	3021 (100.0)
Accra	718 (100.0)	718 (100.0)	0 (0.0)	0 (0.0)	2650 (100.0)
Konongo	103 (100.0)	80 (77.7)	23 (22.3)	0 (0.0)	1098 (100.0)
Larabanga	87 (100.0)	83 (95.4)	3 (3.4)	1 (1.1)	1196 (100.0)
Navrongo	577 (100.0)	575 (99.7)	2 (0.3)	0 (0.0)	795 (100.0)
Paga	173 (100.0)	173 (100.0)	0 (0.0)	0 (0.0)	1765 (100.0)
Total	1895 (100)	1854 (97.8)	40 (2.1)	1 (0.1)	11,506 (100.0)

^a^Raised to adult stage in the insectary

### Blood meal analysis

Blood meal analysis was carried out on blood-fed mosquitoes that were sampled using BG traps and PPK in Larabanga, Navrongo, and Paga. PCR amplification of DNA segments of 20 of 44 blood-fed mosquitoes showed that 18 (90%) had taken a human blood meal, one (5%) had fed on a human and a cow, and one (5%) had taken blood meals from a dog and a goat.

### Phenotypic resistance of *Aedes* to insecticides

**Fig. 4:**
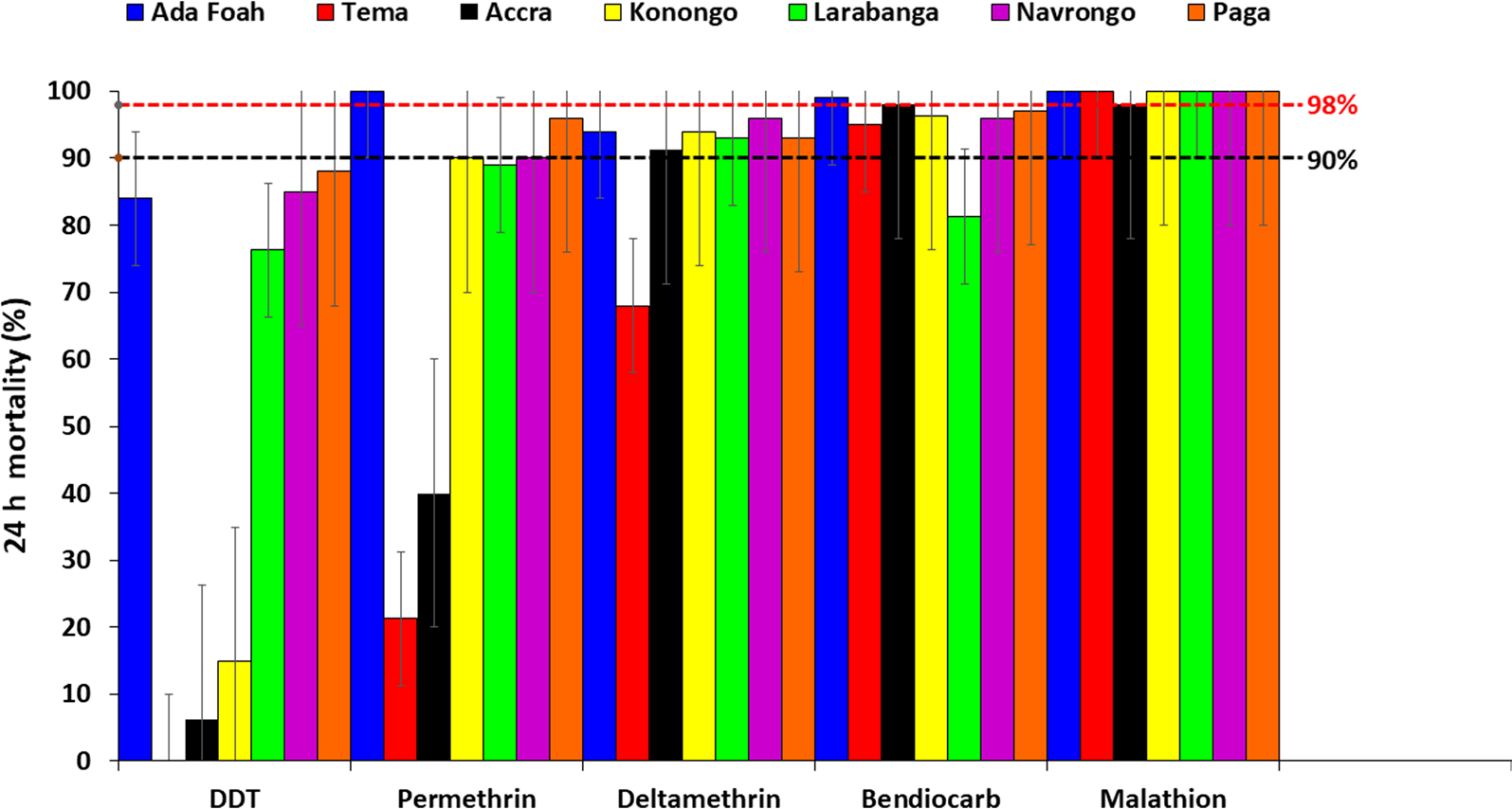
Phenotypic resistance status of *Aedes* mosquitoes to different insecticides.* DDT* Dichlorodiphenyltrichloroethane

Phenotypic test results showed that *Aedes* mosquito populations from all study sites were resistant to DDT (range 0–88%). The highest level of DDT resistance was seen in Tema, where none of the mosquitoes died on exposure to this insecticide. The vectors showed resistance to permethrin in Tema (21%), Accra (40.0%) and Larabanga (89%), and suspected resistance in Navrongo (90%), Paga (96%) and Konongo (90%) ($\chi$ ^2^ = 1.331, *df* = 12, *P* = 0.0001). *Aedes* mosquitoes showed resistance to deltamethrin in Tema (68%) and suspected resistance in Accra (91.3%), Ada Foah (94%), Konongo (94%), Larabanga (93%), Navrongo (96%) and Paga (93%) ($\chi$ ^2^ = 560.000, *df* = 6, *P* = 0.0001). *Aedes* mosquitoes were resistant to bendiocarb in Larabanga (81%)*,* showed suspected resistance in Tema (95.0%), Konongo (96%), Navrongo (96%) and Paga (97%), and were susceptible to bendiocarb in Accra and Ada Foah ($\chi$ ^2^ = 1.331, *df* = 12, *P* = 0.0001). *Aedes* mosquitoes were susceptible to the organophosphate (malathion) at all sites (Fig. 4).

## Discussion

Seasonal variation in population density is common for *Aedes* mosquitoes due to their sensitivity to changes in temperature and moisture [53, 61]. This study found a significantly higher abundance of *Aedes* immatures during the rainy season. The rains may have resulted in an increase in aquatic habitats for *Aedes* spp. that breed in car tyres and the other types of suitable containers that were encountered in this study [62], and thus an increase in the abundance of *Aedes* immatures due to an increase in the rate of oviposition. This finding was similar to that of Ngugi et al. [39], who found a higher abundance of immatures during the rainy season in Kenya. However, Appawu et al. [53] showed an increase in the abundance of *Aedes* immatures during the dry season in Ghana.

Car tyres, buckets, tanks, drums, discarded containers, and air conditioner saucers that had collected water were the main breeding sites and supported the development of *Aedes* immatures in all or some of the study sites. The distribution of *Aedes* immatures between container types varied between the dry and rainy seasons. In all, only car tyres could be considered key breeding habitats in both seasons at all sites, and over 70% of the *Aedes* immatures were collected from these during the study period. The abundance of *Aedes* immatures in car tyres found here is consistent with the findings of a study conducted in the Central African Republic [63], where used car tyres were the most heavily colonized and productive larval habitats for *Aedes* in both early and late wet seasons. Car tyres should therefore be targeted for vector control to eliminate most *Aedes* immatures. Habitats that were non-productive were not included in this study. Larval indices (container index, house index, and Breteau index) were not calculated because we did not record habitats that did not have larvae in them, which was a major limitation of this study.

The WHO susceptibility bioassays showed that *Aedes* mosquito populations from all of the study sites were resistant to DDT. This finding is similar to that of a previous study done in Accra, Ghana, which showed that *Ae. aegypti* were resistant to DDT [64]. Deltamethrin, permethrin, and bendiocarb resistance were also recorded in the present study. As these are some of the most widely used insecticides for the control of *Aedes* spp. [65, 66], their use in Ghana could negatively affect the efficacy of vector control efforts there. Deltamethrin and permethrin resistance could result from the widespread use of pyrethroids for the impregnation of bed nets and for indoor residual spraying against malaria vectors. Pyrethroid resistance has been recently reported in an *Aedes* population in Ghana [64]. Cross-resistance between pyrethroids and DDT is also known to occur [29]. A previous study in Ghana [64] found *Aedes* to be susceptible to permethrin. Other studies have also reported pyrethroid resistance in *Aedes* spp. in Africa and Asia [67–69].

In this study, *Ae. aegypti* was the predominant species in all of the study areas for both adult and larval sampling. The high number of *Ae*. *aegypti* caught from a geographically wide range of sites in different ecological zones may be explained by the behaviour of this species, which is highly anthropophilic and associated with human habitations, as reported by many studies undertaken in Central Africa and West Africa [64, 68, 70]. The high abundance of *Ae*. *aegypti* observed in the present study implies that this vector of yellow fever and dengue is well established in Ghana, which could increase the potential for the transmission of arboviral diseases across the country because outbreaks of these are more severe in the absence of effective vector control.

This calls for constant vector monitoring in Ghana to prevent outbreaks of arboviral diseases. The zoonotic *Aedes* species *Ae*. *africanus* and *Ae. luteocephalus*, which are involved in sylvatic cycles between non-human primates, were also found in this study, which suggests that they may act as bridge vectors and carry disease between sylvan and domestic environments, as both are vectors of yellow fever in Ghana [64]. In addition, Hanley et al. [71] reported that *Ae. africanus* is the primary vector of sylvatic yellow fever virus in the rainforests of Central Africa, extending outward along the riverine forests there, and Diallo et al. [72] detected yellow fever in the zoonotic species *Ae*. *luteocephalus* in Senegal. Suzuki et al. [64] found only one *Ae*. *albopictus* in Accra, Ghana, and in our current study we did not find any individuals of this species in any of the study sites. The absence of this species from our samples suggests that environmental factors in Ghana may not be favourable for its establishment and proliferation. The dominance of its sister species, *Ae*. *aegypti*, may result from favourable environmental conditions for its proliferation.

The present study showed that *Ae. aegypti* rests at an average height of 1.8–2.0 m indoors and 1.3–2.8 m outdoors. These observations are quite different from those of studies undertaken in Iquitos, Peru [58] and Acapulco, Mexico [73], which showed that of 56 and 626 *Ae. aegypti* collected indoors, respectively, 82% rested at a height of less than 1.5 m. Resting height may have major implications for the efficacy of indoor residual spraying due to the exophilic behaviours of some *Aedes* mosquitoes. Insecticide pressure indoors through indoor residual spraying may also trigger exophagy and thus outdoor transmission of arboviruses.

## Conclusions

The results of this study indicate that breeding habitats of *Ae. aegypti* in Ghana are abundant outdoors and are diverse across the country. Car tyres were the most productive containers for *Aedes*, as > 70% of the *Aedes* larvae were collected from these. Thus, targeting tyres in source reduction efforts may be a cost-effective means of reducing the risk of arboviral disease transmission in Ghana. Resistance of *Ae. aegypti *to pyrethroids and carbamates requires careful monitoring as it could limit the efficacy of vector control programmes. Management strategies for vector control that take into account insecticide resistance are thus urgently needed for Ghana.

## Data Availability

All datasets generated and/or analysed during this study are available on request.

## Abbreviations

BG: BG-Sentinel 2
DDT: Dichlorodiphenyltrichloroethane
GPS: Geographical Positioning System
HLC: Human landing catch
PPK: Prokopack Aspirator
WHO: World Health Organization
